# Impact of Obstructive Sleep Apnea on Blood–Brain Barrier Permeability and Membrane Proteins

**DOI:** 10.3390/ijms27156702

**Published:** 2026-07-27

**Authors:** Dominik Jarecki, Szymon Turkiewicz, Piotr Białasiewicz, Dominik Strzelecki, Agata Gabryelska

**Affiliations:** 1 Department of Sleep Medicine and Metabolic Disorders, Medical University of Lodz, 6/8 Mazowiecka St., 92-215 Lodz, Poland; dominik.jarecki@stud.umed.lodz.pl (D.J.); szymon.turkiewicz@umed.lodz.pl (S.T.); piotr.bialasiewicz@umed.lodz.pl (P.B.); 2 Department of Affective and Psychotic Disorders, Medical University of Lodz, 8/10 Czechoslowacka St., 92-216 Lodz, Poland; dominik.strzelecki@umed.lodz.pl

**Keywords:** blood–brain barrier, obstructive sleep apnea, tight junction proteins, VE-cadherin, HIF-1, P-glycoprotein, breast cancer resistance protein, VEGF

## Abstract

The blood–brain barrier (BBB) carefully regulates the transport of molecules between the blood and the nervous tissue. Obstructive sleep apnea (OSA) is a chronic condition that induces structural and functional changes in the BBB. These include alterations in its permeability and in protein expression in the BBB capillary endothelium. Moreover, OSA alters blood levels of transcription factors, such as hypoxia-inducible factor 1 (HIF-1), which are suggested to be responsible for these changes. As a review article, this paper focuses on the most significant effects of OSA on the BBB, including deviations in barrier morphology, particularly changes in the expression of tight junction and adherens junction proteins and membrane channels, as well as their impact on transport across the barrier. Moreover, particular attention is given to emerging evidence for the differential regulation of major BBB efflux transporters, including P-glycoprotein (P-gp) and breast cancer resistance protein (BCRP), as well as the role of the HIF-1 pathway. Additionally, we summarize the current state of knowledge regarding the contribution of these BBB alterations to the development of neurodegenerative diseases. At the same time, altered transport is being investigated as a possibility for delivering new drugs to the central nervous system. Thus, this review aims to summarize the current knowledge about OSA’s impact on the BBB.

## 1. Methods

A literature search was performed using the PubMed database. Articles published up to the end of 2025 were included. The search focused on studies investigating the impact of obstructive sleep apnea (OSA) on the blood–brain barrier (BBB), using keywords such as “OSA”, “intermittent hypoxia”, “BBB”, “tight junctions (TJs)”, “adherens junctions (AJs)”, “Zonula Occludens (ZO)”, and “astrocytes”.

## 2. Blood–Brain Barrier

As the central nervous system (CNS) requires a highly controlled microenvironment to perform its tasks, it must be isolated from external factors, especially substances circulating in the blood. The BBB secures this function. It stops most drugs and exogenous substances from entering the brain and controls the flow of nutrients [1,2]. The BBB consists of endothelial cells, pericytes, and astrocytes.

### 2.1. Endothelial Cells

Endothelial cells are characterized by multiple features that enable them to perform their function in the BBB, mediated by various molecules. The primary structures responsible for the barrier properties are TJs and AJs [3]. Due to their crucial role in maintaining BBB integrity, both TJs and AJs merit individual consideration.

TJs link adjacent endothelial cells, forming an impermeable barrier that restricts the passage of water and solutes [3]. They are composed of transmembrane proteins that connect neighboring cells, including claudins, tricellulin, occludin, and junctional adhesion molecules, as well as auxiliary proteins that anchor these complexes to the cytoskeleton, such as ZO [3]. Interestingly, the regulation of TJs function, essential for BBB maintenance, is not directly mediated by endothelial cells but rather by astrocytes surrounding these vessels [4].

Another essential component of forming this barrier is integrins, which connect the endothelium with the basement membrane [5]. Those proteins, however, are not just structural elements; they also conduct signals both in and out of the cell [6]. For example, integrin connected to laminin promotes BBB maturation and stabilization [6]. There are 24 types of integrin heterodimers, each with a specific ligand, such as extracellular matrix components like laminin or fibronectin [6]. The BBB is not only a static barrier, but it also transports substances into and out of neural tissue through mechanisms including active efflux, receptor-mediated transport (RMT), and carrier-mediated transport (CMT) [5]. Active efflux is executed by ATP-binding cassette (ABC) transporters, efflux pumps powered by ATP, which actively transport xenobiotics and metabolites into the brain vascular lumen [5]. CMT is conducted by proteins encoded by solute carrier (SLC) transporter genes, which allow the uptake of small particles into cells without directly utilizing ATP; however, some of these proteins depend on an ion gradient generated by ATP-powered pumps [5,7]. This type of transport primarily carries nutrients, including carbohydrates, amino acids, nucleotides, organic anions and cations, monocarboxylic acids, fatty acids, vitamins, and choline [5]. Moreover, this type of carrier transports the thyroid hormone triiodothyronine (T3) [5]. RMT is essentially a form of transcytosis in which receptors trigger vesicle formation upon recognizing a specific ligand [8]. Those ligands include arginine-vasopressin, leptin, insulin, apolipoproteins, and β-amyloid [5].

### 2.2. Astrocytes

Astrocytes (ACs), with their foot processes, cover most of the surface of CNS capillaries—around 99% [9]. These processes are characterized by the expression of channels such as aquaporin-4 (Aqp4) and potassium inward-rectifying channel subfamily J member 10 (Kir4.1). Both of those proteins are responsible for regulating cerebral water homeostasis [10]. The brain lacks a lymphatic system, but the recently discovered glymphatic system fills this functional gap [11]. In the glymphatic system, cerebrospinal fluid enters the CNS through the periarterial spaces and is then transported to the interstitium by Aqp4 [11]. This flow drives perivenous drainage of interstitial fluid. It is worth noting that this drainage system can clear waste. During its activity, the nervous system produces waste products, and one of them is β-amyloid [12]—a protein whose accumulation leads to Alzheimer’s disease (AD) [12]. Those waste products are transported to the venous system by interstitial fluid [11], and a negative correlation has been observed between interstitial fluid flow and the severity of AD [13]. Those Aqp4 channels are present on end-foot processes surrounding both arteries and veins [11]. ACs play many roles in the CNS: in addition to the functions mentioned above, they promote the formation and maintenance of synapses and regulate synaptic activity. They also regulate cerebral blood flow and restrict the passage of immune cells across the BBB [9].

### 2.3. Pericytes

**Figure 1:**
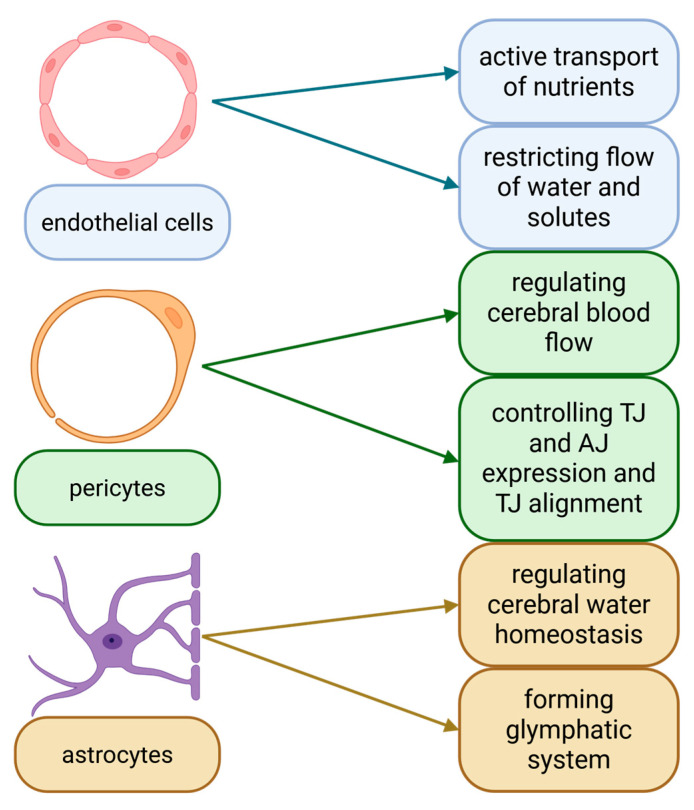
Cell types of the BBB and their function. AJ—adherens junctions; TJ—tight junctions.

Pericytes are a type of cell related to smooth muscle [10], which are connected to the basement membrane of small vessels [14]. They are contractile, allowing them to alter blood flow in capillaries [15]. Moreover, their impact on the BBB includes regulating the expression of TJ and AJ proteins, aligning TJs, and facilitating the transcytosis of fluid-filled vesicles through the BBB [14]. Those cells can communicate with endothelial cells via junctions formed by N-cadherin and connexin-43 hemichannels, which allow the flow of nutrients and signaling metabolites [10]. The summary of all described elements of the BBB is shown in Figure 1.

## 3. Obstructive Sleep Apnea

OSA is a chronic condition characterized by recurrent episodes of upper airway obstruction during sleep [16]. The primary measure of OSA is the apnea-hypopnea index (AHI), defined as the total number of apnea and hypopnea episodes per hour of sleep. Apnea refers to a blockage of airflow lasting at least 10 s [17]. On the other hand, hypopnea is characterized by a reduction in airflow of more than 30% for at least 10 s, followed by a decrease in arterial blood oxygen saturation of more than 3% or arousal [17]. OSA can be diagnosed when AHI ≥ 5; daytime somnolence can also be used as an additional criterion [16]. Furthermore, AHI can be used to define the severity of OSA into the following categories: mild (5 ≤ AHI < 15), moderate (15 ≤ AHI < 30), and severe (AHI ≥ 30) [17]. Polysomnography (PSG) is the gold standard for diagnosing OSA, and the standard treatment is continuous positive airway pressure (CPAP) [18,19], which keeps the pharynx patent [20].

## 4. The Influence of OSA on the BBB

OSA triggers various mechanisms that compromise the BBB. First among those mechanisms is oxidative stress—an imbalance between reactive oxygen species and the cells’ antioxidant capabilities. This imbalance is caused by cycles of deoxygenation and re-oxygenation [21], which, in the context of OSA, is linked with intermittent hypoxia (IH) [22]. IH can be described as recurrent episodes of low tissue oxygenation, separated by periods of normoxia [23]. IH activates nicotinamide adenine dinucleotide phosphate (NADPH) oxidase-dependent production of reactive oxygen species (ROS), which stimulates the synthesis of hypoxia-inducible factor 1α (HIF-1α) and promotes its stability [24]. Another significant change in the organism caused by this disease is the development of an inflammatory state. Although it cannot be directly measured, specific markers can be used to assess it, including C-reactive protein (CRP) [25] and tumor necrosis factor-alpha (TNF-α) [26]. A recent meta-analysis revealed that their elevated levels are both correlated with the presence of OSA and the severity of the disorder [27]. Another OSA effect on the organism is a hypercoagulable state [28]. It is a result of a proinflammatory state mediated by nuclear factor kappa-light-chain-enhancer of activated B cells (NF-κB) [28], whose expression is promoted by HIF-1α [28]—a transcription factor whose serum concentration is elevated in OSA [29]. Moreover, OSA is responsible for chronic sleep fragmentation (SF), which results from multiple micro-arousals [30]. Both SF and IH are main symptoms characterizing OSA [30], which might further cause various complications.

### 4.1. OSA Impact on BBB: In Vitro Studies

At the outset, it is essential to note that OSA in vitro models are predominantly based on the IH paradigm, which serves as the primary experimental framework for investigating the underlying pathophysiological mechanisms.

A comprehensive study using an in vitro BBB model exposed to 6 h of IH, mimicking OSA conditions, revealed significant changes in the BBB’s transcription factors and protein expression, as well as ROS production [31]. TJ and AJ proteins measured in this study included: claudin-5 (CLDN5), vascular endothelial cadherin (VE-cadherin), and zonula occludens-1 (ZO-1). Expression of all these proteins was significantly lower than in the control group [31]. The study also measured efflux pumps [31]. One of them, breast cancer resistance protein (BCRP), showed a 50% decrease in OSA-copycat conditions [31]. Interestingly, P-glycoprotein (P-gp), another transporter, showed a 68% increase in expression. HIF-1α expression doubled in the IH group compared to the control [31]. Similarly, increased HIF-1α protein levels were observed in patients with OSA [19]. Also, nuclear factor erythroid 2-related factor 2 (Nfr2) expression increased by 152% [31], and ROS levels were three times higher after 6 h of IH [31]. Interestingly, Nfr2 increases the expression of genes under the antioxidant response element promoter, such as glutathione S-transferase Ya subunit, thereby potentially protecting the cell from ROS-induced damage [32].

A recent study by Voirin et al. generally confirmed those results. The authors observed increased BBB permeability, decreased expression of CLDN5 and ZO-1, and increased expression of P-gp, as well as other transporters not covered in the previous study, including multidrug resistance protein-1 (MRP-1) [33]. To assess the plausible mediator of these changes, the authors inhibited the HIF-1α pathway using 3-(5′-hydroxyethyl-2′-furyl)-1-benzylindazole (YC-1). Cell cultures treated with YC-1 under IH exhibited protein expression and permeability like those of normoxic cell cultures [33]. This study demonstrated that HIF-1α is involved in BBB changes and suggested that BBB changes in OSA patients may also be mediated by the HIF-1α pathway, as the serum HIF-1α concentration in patients with OSA is approximately twice that in the healthy control group [29].

Interesting conclusions were drawn from a study on the effects of thrombin on the BBB under IH [34]. The authors reported that a low concentration of this clotting factor is protective for the BBB—it lowers BBB permeability and HIF-1α expression during IH [34]. Conversely, higher thrombin concentrations increased permeability and HIF-1α expression [34]. Those findings interfere with the action of a known thrombin inhibitor, dabigatran [34]. In this study, it both inhibited the adverse effects of higher thrombin concentrations and, unfavorably, the protective effects of lower concentrations [34]. These observations encourage further studies into the still-unknown protective mechanisms of thrombin as a potential therapeutic target [34].

### 4.2. OSA Impact on BBB: Trials on Animals

A recent study on a murine model revealed significant changes in barrier permeability after 15 days of IH exposure [35]. Initially, the authors measured BBB permeability using Evans blue dye. It is a dye that was injected into the peritoneum of mice 1 day before brain isolation, and its concentration in brain tissue was subsequently measured [35]. It was demonstrated that, after 15 days of IH, permeability increased approximately twice that of the control group, indicating a profound impact of OSA on the BBB [35]. Moreover, authors observed increased expression of specific BBB-related mRNAs, including aquaporin-1 (Aqp1), aquaporin-9 (Aqp9), occludin, VE-cadherin, and claudin-12 in the dorsal hippocampus [35].

Another study demonstrated that SF in a murine model significantly increased BBB permeability, as measured using dextran–FITC, with increases of approximately 40% after 4 weeks and 50% after 9 weeks compared to the control group [36]. The same study revealed that those changes are fully reversible after about six weeks. The authors also demonstrated a correlation between increased permeability and poorer performance on the novel object recognition test (NOR), providing insight into memory function. Moreover, NOR test results were correlated with BBB permeability—the higher the permeability, the worse the mouse performed in the test [36]. Those observations may correspond with neurological symptoms in humans affected with OSA [37], such as worse emotional memory or higher prevalence of mild cognitive impairment and dementia [38].

### 4.3. OSA Impact on BBB: Clinical Trials

A recent study demonstrated that patients with OSA, both newly diagnosed and those treated with CPAP, exhibit increased BBB permeability [39]. The authors of the study employed a noteworthy method to assess BBB permeability, utilizing the albumin quotient (Qalb)—the ratio of cerebrospinal fluid (CSF) to serum albumin concentrations—as an indirect biomarker of barrier integrity [39]. Higher Qalb values indicate a higher exchange of substances between blood and CSF, suggesting BBB dysfunction. This paper also revealed that newly diagnosed OSA patients have lower CSF β-amyloid42 (Aβ42) levels than healthy subjects and patients on CPAP therapy [39]. This result is consistent with findings from other studies on CSF AD biomarkers in OSA patients, as well as with the correlation between OSA and AD [40]. Lowered Aβ42 in CSF is associated with the presence of amyloid plaques in the brain [41]. Amyloid plaques in the brain serve as reservoirs for soluble Aβ42 peptides, leading to their sequestration within neural tissue. Consequently, despite the overall accumulation of Aβ42 in the brain, its concentration in the CSF decreases due to the limited diffusion of these peptides from the CNS [41]. Considering the previously cited study [39], these findings suggest that amyloid deposition may be more pronounced in OSA patients than in healthy controls, possibly due to BBB dysfunction.

Another study, using diffusion-weighted pseudo-continuous arterial spin labeling (DW-pCASL), a magnetic resonance imaging (MRI) method that detects water flow across the BBB without contrast agents or radioactive tracers, confirmed those observations of increased BBB permeability [42]. Moreover, the authors of this study showed that blood flow in large cerebral arteries did not vary significantly between the OSA and control groups [42]. This observation, as well as previous ones, is consistent with studies on animals and in vitro models, which predicted that IH [30,35], SF [36], and OSA would generally increase BBB permeability.

An overview of the reviewed studies is presented in Table 1.

### 4.4. Limitations of Rodent and In Vitro OSA Models

Studies on rodents and in vitro cell cultures usually utilize IH as a model of OSA [31,35]. However, real-life OSA is a much more complex condition and involves additional phenomena, such as hypercapnia and SF [16,35]. Furthermore, these studies typically involve relatively short periods of exposure to IH, ranging from several hours to a few days, which differs substantially from the chronic nature of OSA in humans [31,33,35]. Taking all of this into consideration, the results of non-human studies should be interpreted with caution, as their outcomes may not fully reflect the mechanisms and manifestations of the disease in clinical settings.

The previously mentioned short duration of rodent and in vitro studies has another important limitation. Their findings may be influenced by short-term effects of IH. For example, in one study, the expression of VE-cadherin was elevated after one day of IH exposure but returned to the previous level after 15 days [35].

### 4.5. Changes in BBB Permeability

Increased BBB permeability was observed across all study types—in vitro, in rodents, and in humans with OSA [33,35,36,39,42]. At this point, we want to synthesize those studies. Despite all those studies exposing increased BBB permeability, their methods, and thus their validity, differ [33,35,36,39,42]. The in vitro study used hypoxia phases lasting 2 h and hydralazine—a compound that induces HIF-1α [33]. Taking this into consideration, this study rather shows how activation of the HIF-1 pathway in OSA affects the BBB than how OSA works [24,33]. It showed that the HIF-1 pathway decreased the number of TJ and AJ proteins and increased BBB permeability, measured by NaFl [33].

Murine models provided experimental conditions closer to OSA, as Roche et al. investigated the impact of 15 days of IH, represented by recurrent episodes of one minute of 5% oxygen followed by one-minute episodes of reoxygenation [35]. Thus, those results are comparable to severe OSA [17]. Puech et al., on the other hand, studied the impact of SF, defined as 30 arousals per hour, on the BBB [36]. Both studies showed increased BBB permeability, measured by the concentration of injected dye in brain tissue [35,36]. Those findings show us that both main symptoms of OSA affect BBB [35,36]. Authors of the study on SF also provided a possible explanation of those permeability changes [36]. They showed that in the brains of mice exposed to SF, microglia get activated [36]. Moreover, in those brains, the concentration of CLDN5 was reduced [36]. Puech et al. explained that microglia, activated by stress factors like SF, activate the NF-κB pathway, resulting in secretion of proinflammatory cytokines like Il-1β and TNF-α, which leads to BBB disruption [36]. Interestingly, the HIF-1 pathway also activates the NF-κB pathway, which may suggest that IH effects are partially explained by microglial secretion of cytokines [28].

Studies on patients with OSA provide data on how the BBB responds to the disease rather than to isolated factors. Both studies showed elevated permeability [39,42]. Interestingly, one of those studies finds that CPAP therapy does not reverse elevated BBB permeability caused by OSA [39]. This result contrasts with a study on SF in a murine model, as that study showed full recovery of BBB function after 6 weeks of normal sleep [36]. It may suggest that mechanisms underlying permanent effects of OSA are linked to IH, rather than to SF.

**Figure 2:**
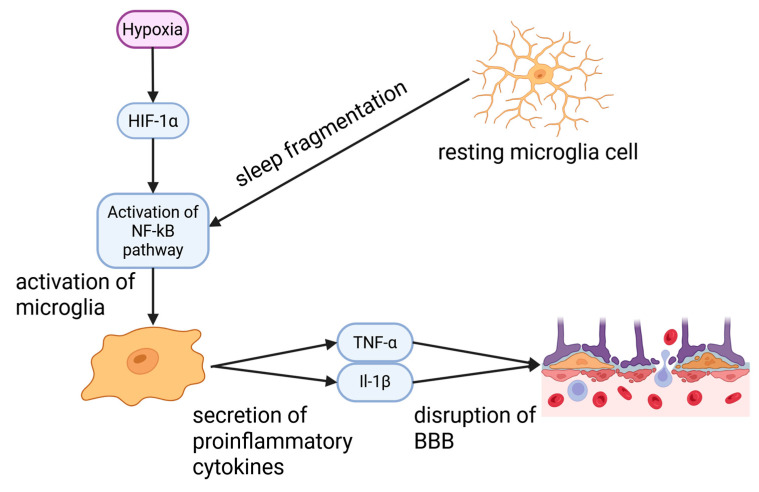
Possible mechanisms of BBB disruption involving microglia following IH and SF. BBB—blood–brain barrier; HIF-1α—hypoxia-inducible factor 1α; IL-1β—interleukin 1β; NF-κβ—nuclear factor κβ; TNF-α—tumor necrosis factor α.

The summary of those mechanisms is presented in Figure 2. 

### 4.6. Changes in Channels in the BBB

Aquaporins are transmembrane channels responsible for the transport of water and small polar molecules. The study by Roche et al. found statistically significant increases in Aqp1 and Aqp9 expression after 15 days of IH [35]. Aqp1, the first identified member of this family, is an example of a classical aquaporin, which passes mostly water [43]. Its overexpression is linked to a significant increase in the viability and mobility of glioma cells [44]. Aqp9 is classified as an aquaglyceroporin and transports particles like arsenic, arsenic trioxide, and 5-fluorouracil [45,46]. As its expression in the brains of mice exposed to 15 days of IH was around 50% higher than in the control group, the effects of particles other than water that access the CNS through it may be significant. One of those particles, which may also be responsible for the positive correlation between the presence of OSA and AD [40], is arsenic, as exposure to this substance was linked with higher morbidity of AD [47] and impairment of neurological functions [48]. In vitro studies on a cholinergic neuron cell line showed that arsenic decreased cell viability; however, interestingly, β-amyloid production was lower than in the control group [49]. Moreover, it has been demonstrated that Aqp9 deletion is protective in a murine model of 1-methyl-4-phenylpyridinium-induced Parkinson’s disease (PD) [50], which is noteworthy because OSA is also associated with a higher prevalence of PD [51].

### 4.7. Changes in Tight Junctions

Claudins form paracellular channels within TJs, and thus allow and control the passage of water, ions, and other solutes [52]. Overexpression of claudin-12 (CLDN12) and occludin was observed in mice exposed to IH [35]. Despite the role of CLDN12 in the BBB remaining unknown [53], mice lacking this protein exhibit reduced locomotor activity and decreased anxiety in females [53]. What is even more interesting is that CLDN12 overexpression was observed in patients with obsessive–compulsive disorder [52], which suggests that it can somehow impact a patient’s mental state. Considering that multiple studies show that OSA is also closely associated with psychiatric disorders, including depression [38,54,55], and dysfunction of the BBB in hypoxic conditions, changes in BBB permeability should be evaluated as a potential pathomechanism of psychiatric complications of OSA.

Another claudin family protein whose expression was altered by IH is CLDN5 [31,35]. CLDN5 is essential for maintaining BBB integrity. Highlighting its critical role, studies in knockout mice demonstrated that complete loss of CLDN5 expression is incompatible with postnatal survival, as none of the animals survived beyond 10 h after birth [56]. In an in vitro study, CLDN5 expression was significantly reduced [30]. At this point, it is worth mentioning that the in vitro study was designed to investigate the isolated effects of HIF-1 pathway activation, whereas the murine study examined the effects of IH [30,35]. It suggests that IH may engage additional regulatory mechanisms that modulate the effects of HIF-1 pathway activation. While no direct comparisons can be made to human studies, it is worth mentioning that in a murine model of OSA, no significant changes in CLDN5 mRNA were revealed [35]. Finally, plasma levels of CLDN5 are higher in patients with AD than in healthy individuals [57]. Interestingly, the same study shows that the CLDN5 level in AD patients, as well as in patients with mild cognitive impairment, gets lower with age. At the same time, this phenomenon does not occur in the healthy population [57]. Moreover, a post-mortem study of AD-affected brains showed that CLDN5 levels were markedly lower than in the control group [58]. Those two facts suggest that underexpression of this claudin may be linked with the later stages of AD. Given the numerous question marks and contradictory information regarding CLDN5, its role in neurodegeneration, and OSA’s impact on its expression, further study is warranted.

Occludin, the first discovered tight junctional protein, is responsible for regulating the BBB’s permeability. Moreover, it regulates the transepithelial migration of neutrophils [7,59]. Given its elevated expression in the murine model of IH [35], it is essential to consider its role in OSA, particularly considering other disease effects that interfere with this protein. For example, vascular endothelial growth factor (VEGF), whose plasma levels in patients with OSA are significantly higher than in normal individuals [60], induces occludin phosphorylation, thereby increasing water permeability in an in vitro BBB model [61]. Yet, other particles, such as interferon-ɣ (IFN-ɣ), regulate the BBB through occludins [62]. In vitro studies have shown that IFN-ɣ rapidly increases BBB permeability [63]. It is important to note that current research on IFN-ɣ levels in adult patients with OSA is insufficient to determine whether these levels differ from those observed in healthy individuals [64].

In contrast, elevated IFN-ɣ concentrations have been reported in pediatric patients with OSA [64]. Also, some neurodegenerative diseases correlate with elevated levels of occludin. Postmortem study of brains of humans with AD and vascular dementia (VD) showed significantly elevated levels of occludin compared to the control group [65], which once more shows that OSA and AD might provoke similar reactions in the brain.

The last tight-junctional protein whose expression was altered in IH is ZO-1 [30]. In vitro, its function is well understood—it links occludin to the cell’s actin cytoskeleton [66]. However, its in vivo function remains unclear [66]. Interestingly, changes in its expression differ across sleep disorders. The IH model shows reduced ZO-1 levels [31], whereas in insomnia disorder, ZO-1 levels are significantly increased [67]. Perhaps this increase is related to the previously mentioned glymphatic system, as its function correlates with the state of consciousness [11]; however, this is only speculation. Yet, no studies have examined how ZO-1 expression in the BBB changes in patients with OSA.

### 4.8. Changes in Adherens Junctions

VE-cadherin is a key protein mediating cell-to-cell adhesion within the vascular endothelium. It interacts with VE-cadherin molecules on neighbouring endothelial cells and anchors to the intracellular cytoskeleton through associations with β-catenin, plakoglobin (also known as γ-catenin), and p120-catenin [68]. Its importance for vascular integrity is underscored by the fact that VE-cadherin knock-out mice die at an early embryonic stage [68]. Downregulation of this protein has been shown to increase the permeability of the brain microvascular endothelium [69].

In an in vitro model of IH, VE-cadherin expression was significantly reduced [31]. In contrast, in a murine model, its expression was markedly elevated after one day of IH exposure, but not after 15 days [35]. Interestingly, serum VE-cadherin levels in patients with OSA were significantly higher than those in healthy controls [70]. The authors of that study suggested that elevated circulating VE-cadherin levels reflected damage to the brain capillary endothelium, with protein fragments released into the bloodstream [70]. They further associated these findings with increased BBB permeability and reduced VE-cadherin expression in the brain [70].

As these are currently the only available studies addressing VE-cadherin levels in relation to BBB integrity in OSA, and their results remain inconclusive regarding the precise response of this protein to OSA, further research is necessary.

### 4.9. Changes in the Expression of Transcription and Growth Factors

As transcription factors regulate DNA expression to adjust the organism to changing conditions, it is evident that OSA, with episodes of arterial blood oxygen desaturation, alters their levels; multiple studies have observed significant changes in their expression [31,71,72].

The first of the described transcription factors, HIF, is a dimer consisting of alpha and beta subunits [73]. The most well-known subunit is HIF-1α [73]. HIF-1 is a protein that accumulates in the cytoplasm and translocates to the nucleus under hypoxic conditions, activating transcription of genes such as VEGF, erythropoietin (EPO), induced nitric oxide synthase (iNOS), and the glucose transporter (GLUT) [73]. The broad range of HIF-1 effects encompasses both local responses (GLUT, VEGF) and organismal adjustments (EPO). Although this transcription factor alters gene expression to maintain homeostasis, increased iNOS expression, leading to nitric oxide (NO) production, can cause neuronal death, especially when combined with hypoxic conditions [74], which are characteristic of OSA. The previously mentioned cell culture study found a twofold increase in expression under IH conditions [30]. No studies have measured HIF-1 levels in the human brain or CSF of OSA patients, but serum HIF-1 levels have been measured and found to be significantly elevated [71]. As previously noted, inhibition of the HIF-1 pathway prevents changes in the BBB under IH [33]. The authors of that study suggested that HIF-1 inhibitors might be considered a treatment for hypoxia-induced cognitive disorders [33]. Because OSA causes neurological deficits [40], this opportunity should be explored.

Under IH conditions, elevated VEGF levels have been observed [72]. The upregulation of HIF-1 can explains this effect, as VEGF is one of the target genes activated by this transcription factor [73]. Overexpression of VEGF has been shown to increase BBB permeability [75], which in turn may contribute to the development of brain edema [76]. Interestingly, the same study demonstrated a paradoxical protective effect of increased VEGF expression in a murine model of ischemic stroke [75]. Specifically, VEGF upregulation has been shown to attenuate neurological deficits [75]. This effect was attributable not only to increased vascular density but also to remodeling of cerebral blood flow. In the ischemic core, perfusion was significantly reduced, while it normalized in the peripheral regions and increased in surrounding tissues [75]. These findings are particularly intriguing, given that OSA—where VEGF is consistently overexpressed—is recognized as an independent risk factor for stroke [77]. In addition, VEGF overexpression in a proinflammatory environment leads to increased BBB permeability, exposure of cerebral antigens, and enhanced leukocyte adhesion to the endothelium [76]. Such processes may contribute to microglial activation, a hallmark feature of OSA-related neuropathology [78].

Taken together, the role of VEGF in OSA appears highly complex: while its upregulation may mediate neuroprotective effects under ischemic conditions, its contribution to BBB disruption, inflammation, and microglial activation suggests a dual, context-dependent influence that warrants further elucidation.

### 4.10. Changes in Drug Transport

As OSA increases BBB permeability and induces overexpression of selected transmembrane transporters, its potential impact on pharmacokinetics requires careful consideration. Under physiological conditions, only small lipid-soluble molecules and compounds with specific membrane transporters can cross the BBB. Depending on the study, data suggest that only 6% to 12% of pharmacological agents exert activity within the CNS, and after excluding drugs designed for affective disorders, the number falls to only 1% [2].

One such compound is 5-fluorouracil (5-FU) [79]. As noted earlier, 5-FU utilizes Aqp9 channels to enter the brain, and the abundance of these channels is significantly upregulated in mice exposed to chronic IH [35]. Consequently, OSA may influence both the therapeutic efficacy and adverse effects of 5-FU; however, to date, no studies have specifically addressed this issue.

Critical transporters involved in drug disposition include P-gp, an ATP-dependent efflux pump located on the luminal surface of cerebral capillary endothelial cells [80]. P-gp actively extrudes both endogenous and exogenous substrates from the CNS [80].

Overexpression of P-gp has been demonstrated not only in in vitro BBB models exposed to IH [30] but also in rodents subjected to IH [81]. Although that study assessed P-gp levels in myocardial arteries and veins, no other in vivo investigations are currently available. Considering that both the BBB and myocardial vessels are exposed to the same systemic circulation, similar endothelial alterations in the BBB are likely [81]. This suggests that P-gp upregulation may represent a compensatory mechanism to limit the penetration of circulating xenobiotics or inflammatory mediators into the brain. Still, it could also impair the transport of endogenous substrates across the BBB, potentially exacerbating the neurovascular dysfunction observed in patients with OSA. Clinically, this protein plays a crucial role in the treatment of high-grade gliomas (HGGs). Vincristine, the most effective chemotherapeutic agent for this tumor type [82], is actively transported from the CNS by P-gp [83]. This markedly reduces its concentration in glioma tissues. If OSA similarly increases P-gp expression in humans, as suggested by experimental data, this remains an important hypothesis that should be further investigated, as it may open new therapeutic possibilities in patients with this condition. Notably, P-gp activity can be pharmacologically inhibited by verapamil [83], a widely used cardiovascular medication, which raises the possibility of therapeutic modulation.

Another efflux transporter expressed at the BBB is breast cancer resistance protein (BCRP). Unlike P-gp, BCRP expression is significantly downregulated under in vitro IH conditions [30]. BCRP is involved not only in drug transport but also in neurodegenerative processes [84]. In BCRP knock-out mice, β-amyloid accumulation—a pathological hallmark of AD—was markedly increased [84]. This finding suggests that OSA-related alterations in BCRP expression may contribute to neurodegenerative risk, thereby adding to the mechanisms previously linked to changes in BBB permeability.

**Figure 3:**
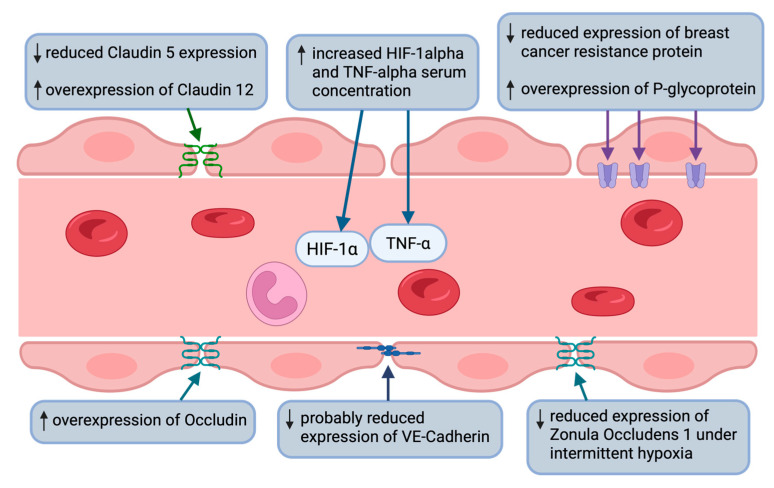
The summary of changes in the BBB caused by OSA, IH, and SF. HIF-1alpha—hypoxia-inducible factor 1 alpha; IH—intermittent hypoxia; OSA—obstructive sleep apnoea; SF—sleep fragmentation; TNF-alpha—tumor necrosis factor alpha; VE-cadherin—vascular endothelial cadherin.

**Figure 4:**
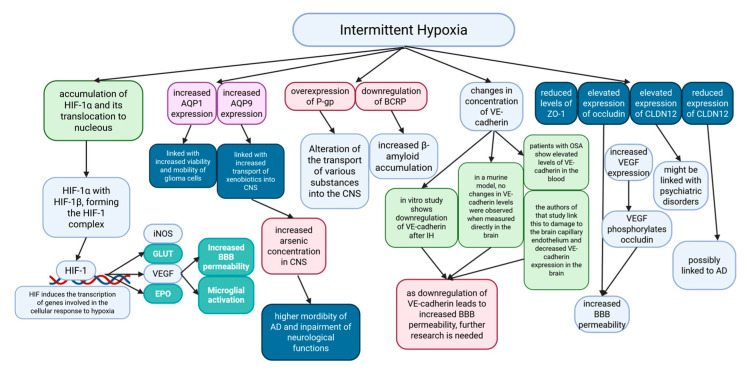
Summary of IH effects on the BBB. AD—Alzheimer disease; AQP1—aquaporin 1; AQP9—aquaporin 9; BBB—blood–brain barrier; CLDN12—claudin 12; CNS—central nervous system; EPO—erythropoietin; GLUT—glucose transporter; HIF-1alpha—hypoxia-inducible factor 1 alpha; IH—intermittent hypoxia; iNOS—inducible nitric oxide synthase; VE-cadherin—vascular endothelial cadherin; VEGF—vascular endothelial growth factor; ZO-1—Zonna Occludens 1.

The summary of changes in the BBB and the effects of IH on the BBB in OSA are shown in Figure 3 and Figure 4.

## 5. Conclusions

It has been demonstrated that OSA alters BBB properties through multiple mechanisms. Both IH and SF affect the expression of junctional proteins in the endothelial layer of the BBB, as well as the regulation of efflux pumps and transcription factors. Most of these alterations directly contribute to increased BBB permeability, which is broadly consistent with experimental observations. In addition, IH and SF may promote neurodegenerative processes, which is in line with epidemiological evidence linking OSA to an increased risk of disorders such as AD. Nevertheless, the underlying mechanisms remain complex and are only partially elucidated. Notably, many studies investigating changes in protein expression have been conducted exclusively in animal or in vitro models, leaving considerable uncertainty regarding the extent to which these findings can be applied to humans.

Given that the BBB physiologically restricts the entry of most pharmacological agents into the CNS, it is also important to note that altered expression of channel proteins and efflux pumps, as well as increased overall BBB permeability, may significantly affect drug pharmacokinetics. Although this area of research remains underdeveloped, emerging studies suggest that such changes might eventually be exploited to enhance CNS drug delivery.

In summary, OSA-induced modifications in BBB integrity not only provide a plausible link between sleep-disordered breathing and neurodegeneration but also have potential implications for therapeutic strategies, both as a challenge to treatment safety and as a possible avenue for improved drug delivery.

## Figures and Tables

**Table 1:** Overview of the reviewed studies. Aβ42—β-amyloid42AQP1—aquaporin 1; AQP9—aquaporin 9; BBB—blood–brain barrier; BCRP—breast cancer resistance protein; CLDN5—claudin 5; CPAP—continuous positive airway pressure; CSF—cerebro-spinal fluid; HIF-1—hypoxia-inducible factor 1; IH—intermittent hypoxia; MRP-1—multidrug resistance-associated protein 1; Nfr2—nuclear factor erythroid 2-related factor 2; OSA—obstructive sleep apnoea; P-gp—glycoprotein P; SF—sleep fragmentation; ROS—reactive oxygen species; VE-cadherin—vascular endothelial cadherin; ZO-1—Zonna Occludens 1.

Main Findings	Exposure	Model	Study
CLDN5, ZO-1, VE-Cadherin and BCRP expression decrease; HIF-1α, P-gp and Nfr-2 expression increase; 3 times higher ROS levels	IH	In vitro	Zolotoff et al. [31]
Increased BBB permeability; CLDN5 and ZO-1 expression decrease; P-gp and MRP-1 expression increase; Those changes did not occur in the IH group with inhibited HIF-1 pathway	IH	In vitro	Voirin et al. [33]
Increased BBB permeability; Aqp1, Aqp9, occludin and VE-Cadherin expression increase	IH	Murine	Roche et al. [35]
Increased BBB permeability; Poorer performance in NOR test	SF	Murine	Puech et al. [36]
Increased BBB permeability, also after CPAP therapy; Lower CSF Aβ42 levels	OSA, CPAP	Human	Fernandes et al. [39]
Increased BBB permeability	OSA	Human	Palomares et al. [42]

## Data Availability

This manuscript is a review article. All data supporting the findings presented in this review were obtained from publicly available published literature. No new datasets were generated or analyzed during the current study. For any additional information regarding the data sources or study selection, please contact the corresponding author.
